# Surface display of p75, a *Lactobacillus rhamnosus* GG derived protein, on *Bacillus subtilis* spores and its antibacterial activity against *Listeria monocytogenes*

**DOI:** 10.1186/s13568-020-01073-9

**Published:** 2020-08-08

**Authors:** Soo Ji Kang, Ji Su Jun, Jeong A Moon, Kwang Won Hong

**Affiliations:** Department of Food Science and Biotechnology, College of Life Science and Biotechnology, Dongguk University, Goyang-si, 10326 Republic of Korea

**Keywords:** Spore surface display, p75 protein, Antibacterial activity, *L. monocytogenes*

## Abstract

*Lactobacillus rhamnosus* p75 protein with peptidoglycan hydrolase (PGH) activity is one of the key molecules exhibiting anti-apoptotic and cell-protective activity for human intestinal epithelial cells. In this study, with the goal of developing new probiotics, the p75 protein was displayed on the surface of *Bacillus subtilis* spores using spore coat protein CotG as an anchoring motif. The PGH activity, stability, and the antibacterial activity of the spore-displayed p75 (CotG-p75) protein were also investigated. The PGH activity of the CotG-p75 against peptidoglycan extracted from *B. subtilis* was confirmed by the ninhydrin test. Under various harsh conditions, compared to the control groups, the PGH activities of CotG-p75 were very stable in the range of pH 3–7 and maintained at 70% at 50 °C. In addition, the antibacterial activity of CotG-p75 against *Listeria monocytogenes* was evaluated by a time-kill assay. After 6 h incubation in phosphate-buffered saline, CotG-p75 reduced the number of viable cells of *L. monocytogenes* by up to 2.0 log. Scanning electron microscopy analysis showed that the cell wall of *L. monocytogenes* was partially damaged by the treatment with CotG-p75. Our preliminary results show that CotG-p75 could be a good candidate for further research to develop new genetically engineered probiotics.

## Introduction

*Lactobacillus rhamnosus* GG (LGG) is one of the most widely studied probiotic strains and has been reported to be effective in treating and preventing ulcerative colitis, diarrhea, and atopic dermatitis in numerous clinical studies (Zocco et al. 2006; Szajewska et al. 2011; Doron et al. 2005). p75, also known as major secreted protein 1, is one of the soluble proteins derived from LGG and is a key molecule that shows probiotic features. p75 regulates the proliferation and survival of intestinal epithelial cells through stimulation of Akt activation, inhibition of cytokine-induced epithelial cell apoptosis, promotion of cell growth, and reduction of tumor necrosis factor (TNF)-induced colon epithelial damage (Yan and Polk 2002; Yan et al. 2007). In addition, p75 protects the intestinal epithelial tight junctions and barrier functions from disruption by hydrogen peroxide (Seth et al. 2008). On the other hand, many studies have shown that p75 has sequence homology with peptidoglycan hydrolase (PGH) and that has d-glutamyl-l-lysyl endopeptidase activity (Bäuerl et al. 2010; Claes et al. 2012). However, it is not yet known whether the PGH activity of p75 is directly related to its various probiotic properties mentioned above.

Although probiotics have numerous health benefits including treating and preventing intestinal disease, they have limitations. Because probiotic action is non-specific and non-discriminatory, it is affected by strains or dose, and the effect may vary from person to person (Bomba et al. 2002; Karimi and Peña 2008; Morrow and Kollef 2008). Since probiotics are living microorganisms, they tend to have low viability during food processing and passage through the gastrointestinal tract (Mattila-Sandholm et al. 2002). To overcome these limitations, there have been many efforts to develop new probiotics through bioengineering. To improve stress tolerance, the betaine transporter gene (*betL*) of *L. monocytogenes* and the trehalose synthesis gene (*ostAB*) of *Escherichia coli* were cloned into probiotic strains of *Lactobacillus* and *Bifidobacterium* (Sheehan et al. 2006; Termont et al. 2006; Sheehan et al. 2007). In addition, probiotic strains and *E. coli* were engineered to produce antimicrobial peptides or compounds to enhance its antimicrobial actions (Goh et al. 2012; Chen et al. 2010).

Spore surface display technology has been proposed as an alternative approach to develop new probiotics with improved stability and efficiency. Spore surface display is a technique for displaying foreign proteins on the endospores of spore-forming bacteria. *B. subtilis,* a non-pathogenic spore-forming bacteria, has been widely used for spore surface displays due to its GRAS (general recognized as safe) status and probiotic features (Elshaghabee et al. 2017; Cutting 2011; Hong et al. 2005)*. B. subtilis* spores can survive in extreme environments due to its unique structure. It is encased in a thick peptidoglycan layer and many coat proteins (Henriques and Moran 2007; Nicholson et al. 2000). With these properties, spore displayed proteins are more stable than free ones (Zhang et al. 2019; Chen et al. 2017; Kim and Schumann 2009). Therefore, spore surface display has been applied in various fields such as vaccine and drug development, whole-cell biocatalyst, and multimeric protein production (Zhang et al. 2019). Recently, it has been actively applied in the food industries for production of materials such as d-neuraminic acid (Xu et al. 2011), d-tagatose (Guo et al. 2018; Liu et al. 2014) D-allulose (He et al. 2016), lactulose (Wang et al. 2016), and trehalose (Liu et al. 2019). However, it has not been previously reported that spore surface display technology has been applied to the development of new probiotics.

In this study, we displayed the p75 protein on the surface of *B. subtilis* spores using CotG as an anchoring motif to develop a novel probiotic. To confirm the successful expression of the displayed p75 (CotG-p75), the PGH activity of CotG-p75 against peptidoglycan was examined. In addition, to investigate the stability of CotG-p75 on the spore surface, the PGH activity of CotG-p75 was measured under various temperature and pH conditions. Finally, the antibacterial effect of CotG-p75 on Gram positive bacteria was investigated for *L. monocytogenes*, a major food poisoning bacterium.

## Materials and methods

### Bacterial strains, growth conditions, and transformation

The bacterial strains used in this study are listed in Table 1. *E. coli* DH5α was used for the subcloning experiment and transformation. *B. subtilis* 168 was used as the host strain to display the p75 protein on its endospore surface. *E. coli* and *B. subtilis* were routinely grown in Luria-broth (LB) medium at 37 °C in a shaking incubator. *L. rhamnosus* GG ATCC 53103 was used to obtain the p75 gene and it was cultured in Man-Rogosa-Sharpe (MRS) medium at 37 °C. *Listeria monocytogenes* ATCC 19115 was cultured in Brain Heart Infusion (BHI) broth at 37 °C in a shaking incubator. In addition, polymyxin acriflavine lithium chloride ceftazidime aesculin mannitol agar was used as a selective medium for *L. monocytogenes* in the time-kill assay. The transformation of *E. coli* DH5α used the CaCl_2_-mediated transformation method described elsewhere (Sambrook et al. 1989). The recombinant plasmid was transformed into *B. subtilis* 168 by the method of Juhas and Ajioka (2016). Antibiotics, ampicillin (50 µg/mL) and kanamycin (10 µg/mL), were added into the medium for the selection of the *E. coli* and *B. subtilis* cells harboring the pUB19-*cotG*-*p75*, respectively.

**Table 1:** Bacterial strains, plasmids, and primers used in this study

Bacterial strains or plasmids	Description	Reference
Bacterial strains		
*Escherichia coli* DH5α	*F*−*, φ 80dlacZ∆M15, ∆(lacZYA*-*argF)U169, deoR, recA1, endA1, hsdR17(rK*−*, mK*+*), phoA, supE44, λ*−*, thi*-*1, gyrA96, relA1*	Hanahan (1983)
*Bacillus subtilis* 168	*trpC2*	Anagnostopoulos and Crawford (1961)
*Lactobacillus rhamnosus* GG	ATCC 53103	Purchased from ATCC
*Listeria monocytogenes*	ATCC19115	Purchased from ATCC
Plasmids		
pUB19	*E. coli*-*B. subtilis* shuttle vector, Ap^r^, Km^r^	Kang et al. (2019)
pUB19-*cotG*-*p75*	Spore display of p75 using the CotG anchor	This study
Primers		
CotG-F	5′-CTTCG**ACGCGT**CAGCTGGCACC-3′	This study
CotG-R	5′-GTCCCTGTCGAGCTTCCTCCTCCTCCTTTGTATTTCTTTTTGACTACCCAGCAATTGCCGT-3′	This study
P75-F	5′-AGAAATACAAAGGAGGAGGAGGAAGCTCGACAGGGACGGTCAGTTACAAATCCGG-3′	This study
P75-R	5′-AAGGAAAAA**GCGGCCGC**TTATAGTGACGGGCGAACCGCAAAGTCAGG-3′	This study

^a^ ATCC, American type culture collection (Manassas, VA, USA)

^b^ The bold letters indicate the restriction sites

^c^ The underlined letters indicate a flexible linker (GGGGS) at the C-terminal of the *cotG* structural gene

### Plasmid construction

The primers and plasmid used in this study are listed in Table 1. The *cotG* gene, containing its promoter region and the protein coding region, was amplified by polymerase chain reaction (PCR) using the *B. subtilis* 168 chromosome as a template and primer sets of cotG-F/cotG-R. The DNA fragment containing the *p75* structural gene except 96 bp of the signal peptide sequence was amplified with primer sets p75-F/p75-R using the *Lactobacillus rhamnosus* GG (LGG) chromosome as a template. Fragments of the amplified *cotG* and *p75* genes were fused by overlap extension PCR with oligonucleotide pair cotG-F/p75-R. A flexible linker (Gly-Gly-Gly-Gly-Ser) was inserted between the C-terminus of the CotG protein and the N-terminus of the p75 protein to improve the structural flexibility of the fused protein (Wriggers et al. 2005). The amplified *cotG*-*p75* DNA (2264 bp) was digested with *Mlu*I and *Not*I restriction enzymes and ligated into the shuttle vector pUB19 (Km^r^) digested with the same enzymes. The resulting plasmid was named pUB19-*cotG*-*p75*.

### Preparation of spores

The recombinant *B. subtilis* 168 containing the pUB19-*cotG*-*p75* was cultured for 62 h at 37 °C in Difco Sporulation Medium (DSM) in an incubator shaking at 150 rpm. Spores were harvested and prepared as described previously (Nicholson and Setlow 1990). The spores were resuspended in 50 mM sodium phosphate buffer (pH 7.2) and treated with lysozyme at a final concentration of 0.05% for 1 h at 4 °C to destroy any residual vegetative cells. Then the spores were washed 5 times with 50 mM sodium phosphate buffer to remove any residual lysozyme. The colony forming units (CFU) of the spores were measured by plating serial dilutions on LB agar medium containing kanamycin (10 µg/mL). The final concentration of spores in the buffer was adjusted to 10^9^ CFU/mL.

### Preparation of peptidoglycan

Peptidoglycan (PG) was prepared from the mid-log phase of *B. subtilis* as described previously by Atrih et al. (1999) with a slight modification. Bacterial cell cultures (250 mL) were boiled and then centrifuged at 14,000×*g* at 4 °C for 8 min. The pellet was resuspended in 5% (W/V) hot sodium dodecyl sulfate (SDS) and boiled for 25 min. After centrifugation at the same condition, the pellet was resuspended in 4% (W/V) SDS and boiled for 15 min. The insoluble material was collected by centrifugation and washed with distilled water (DW) more than six times to eliminate all of the SDS. Proteinase K (2 mg/mL) and trypsin (200 μg/mL) were added for 1 h and 16 h, separately, at 37 °C to remove covalently attached proteins. Then the insoluble material was recovered by centrifuge and treated in 48% (V/V) hydrofluoric acid for 24 h at 4 °C. After recovery by centrifugation, the insoluble cell wall was resuspended in 50 mM Tris–HCl buffer (pH 7) and washed with cold DW at least five times to adjust it to a neutral pH. Then, the PG extract was suspended in 1.5 mL DW and stored at 4 °C.

### PGH activity assay of CotG-p75

Identification of the PGH activity of CotG-p75 was carried out using the ninhydrin method and the *B. subtilis* PG extract as the substrate (Moore and Stein 1948). In brief, 30 µL of CotG-p75 at different concentrations (1.2, 1.8, 2.4, 3.0, and 3.6 × 10^5^ CFU/mL) was mixed with 30 µL of PG extract. After 15 min incubation at 37 °C, the mixtures were centrifuged at 13,000 rpm for 5 min to precipitate any unhydrolyzed PG extract. Then, 30 µL of the supernatant was placed in a new tube and reacted with 3 µL of 2% (W/V) ninhydrin solution. The reaction solution was heated for 5 min at 100 °C and then cooled to room temperature. The absorbance of the reaction mixture was measured at 570 nm with a Nanodrop 2000 spectrophotometer (Thermo Fisher Scientific, Wilmington, DE, USA). PG treated with DW instead of CotG-p75 was used as a blank. CotG-p75 reacted with DW instead of PG extract was used as the control. The enzymatic activity was measured three times with independent samples.

### Stability test

The stability of CotG-p75 was determined under different temperature and pH conditions by the ninhydrin method. To identify its thermal stability, 30 µL of spores (3.6 × 10^5^ CFU/mL) were heated at each temperature (40, 50, 60, 70, and 80 °C) for 15 min using a heating block. Then, all samples were cooled to room temperature (25 °C). To evaluate its pH stability, 30 µL of spores were treated with Mcilvaine’s buffer (pH 2, 3, 4, 5, 6, 7, and 8) for 15 min. Then all samples were washed three times with phosphate-buffered saline (PBS, pH 7.4). After each treatment, the sample was incubated with the PG extract at 37 °C for 15 min. Then, the PGH activity of each sample was measured with the ninhydrin method as described above. The relative PGH activity was calculated by defining the activity measured at 25 °C and pH 7 as 100%.

### Antibacterial evaluation

The antimicrobial effect of CotG-p75 was identified using a time-kill assay based on the National Committee for Clinical Laboratory Standards guidelines (Barry et al. 1999). To identify the antimicrobial effect of CotG-p75 against pathogenic Gram-positive bacteria, *L. monocytogenes* ATCC 19115 was used. *L. monocytogenes* was grown to the log phase in BHI broth at 37 °C. The cells were harvested and washed three times with PBS. The cell pellets were resuspended and adjusted to 10^8^ CFU/mL with PBS. The time-kill assay was performed in PBS where spores could not germinate and the pathogen could not grow during the incubation period.

To test the antibacterial activity of CotG-p75 against *L. monocytogenes*, the ratio of CotG-p75 to *L. monocytogenes* was adjusted to 0.1, 1, and 10. One mL each of the *L. monocytogenes* (10^8^ CFU/mL) and CotG-p75 (10^7^, 10^8^, and 10^9^ CFU/mL) were mixed together in a 5 mL Eppendorf tube and incubated at 37 °C for up to 6 h. Wild-type spores and PBS with no spores were mixed with 10^8^ CFU/mL of *L. monocytogenes* and used as controls. During the incubation, 0.1 mL of each culture was taken at 1 h intervals, diluted as indicated, and then plated on selective medium for *L. monocytogenes*. At the same time, a light microscope (Olympus-CX31, Olympus, Tokyo, Japan) was used to roughly observe the change in the number of *L. monocytogenes* cells in each sample (Additional file 1: Fig. S1). The number of viable *L. monocytogenes* cells was counted after 24–48 h incubation on selective medium at 37 °C. The log reduction was calculated using the following formula: Log reduction = log10 (number of viable cells before treatment/number of viable cells after treatment).

### Scanning electron microscopy (SEM) analysis

For SEM analysis, spores were purified using a Renografin gradient method to exclude vegetative cells of *B. subtilis*. *B. subtilis* 168 harboring pUB19-*cotG*-*p75* was cultured for 120 h and harvested by centrifugation at 5000×*g* for 10 min. The collected pellet was resuspended in 20% sodium diatrizoate, and then the suspension was layered over 50% sodium diatrizoate. After centrifugation at 10,000×*g* for 30 min, the purified spores were collected at the bottom of the tube. The spores were washed with PBS at least three times to remove any residual sodium diatrizoate.

Sample preparation for SEM was carried out as described by Kockro et al. (2000) with slight modifications. Broth cultures of *L. monocytogenes* ATCC 19115 (10^8^ CFU/mL) were washed three times with PBS. An aliquot of *L. monocytogenes* (100 μL) was treated with 100 μL of CotG-p75 (10^8^ CFU/mL). After incubation at 37 °C for 4 h, the specimen was fixed in 2.5% (V/V) glutaraldehyde for 2 h at 4 °C. The fixed cells were dehydrated through a gradient of 35, 50, 75, 95, and 100% ethanol for 10 min each. The dehydrated cells were dried using hexamethyldisilazane (HMDS). Then, the dried cells were coated with gold using a sputter coater (108 auto sputter coater, Cressington Scientific Instruments, Watford, UK). The samples were then observed under a scanning electron microscope (SEM, JSM-7800F Prime from JEOL Ltd., Tokyo, Japan).

### Statistical analysis

All data presented in this study are expressed as mean ± standard deviation from triplicate samples. The statistical significance of differences between groups was determined by an unpaired two-tailed t-test using GraphPad Prism 5 (GraphPad Software Inc., La Jolla, CA, USA) at a significance level of *p* < 0.05.

## Results

### Construction of the recombinant plasmid

**Fig. 1:**
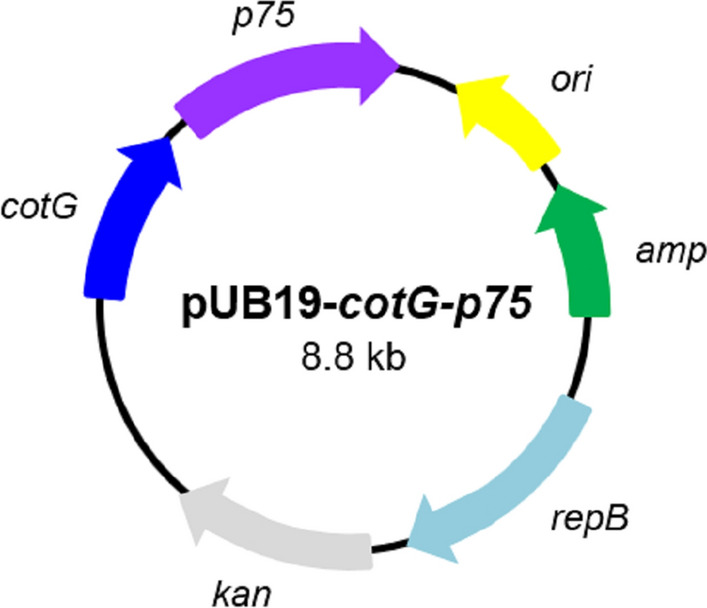
Plasmid map of the recombinant plasmid pUB19-*cotG*-*p75*. The *ori* and *rep*B represent the replication origin and replication protein B, respectively. The *amp* and *kan* represent ampicillin and kanamycin resistance markers, respectively. The *cotG* and *p75* represent the spore coat protein CotG encoding gene of *B. subtilis* and the p75 protein encoding gene of *L. rhamnosus* GG, respectively

The recombinant pUB19 plasmid containing the *cotG* and *p75* genes was constructed and verified by restriction enzyme analysis and PCR (Additional file 1: Fig. S2). The recombinant plasmid was named pUB19-*cotG*-*p75* and a diagram of its structure is shown in Fig. 1. The spores displaying the fusion protein obtained from *B. subtilis* harboring pUB19-*cotG*-*p75* were designated as CotG-p75.

### Peptidoglycan hydrolase activity of CotG-p75

**Fig. 2:**
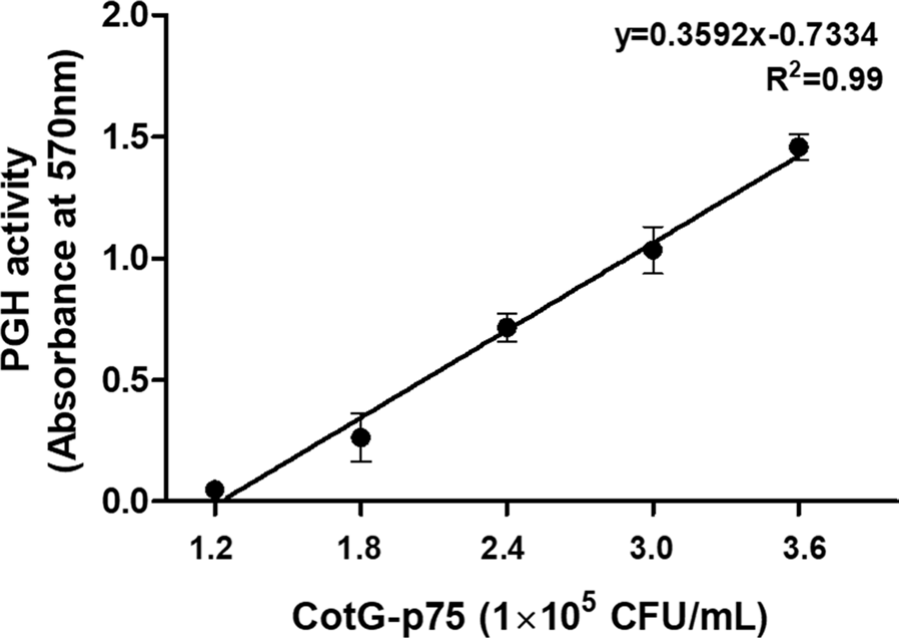
Determination of the peptidoglycan hydrolase (PGH) activity of CotG-p75 measured by the ninhydrin method. After treatment of peptidoglycan with different concentrations of CotG-p75 at 37 °C for 15 min, the absorbance of each sample was measured at 570 nm. All tests were performed in triplicate, and the data are presented as mean ± standard deviation

A ninhydrin test was performed to verify that the CotG-p75 displayed on the spore surface maintained its intrinsic PGH activity. PG was treated with different concentrations (1.2, 1.8, 2.4, 3.0, and 3.6 × 10^5^ CFU/mL) of CotG-p75 and the degree of PG degradation was measured by the ninhydrin test. As shown in Fig. 2, the absorbance increased linearly as the concentration of CotG-p75 increased (R^2^ = 0.99). The PGH activity of the p75 protein fused to the CotG protein seems to be well maintained. Thus, the fusion of the p75 protein with the CotG protein located on the spore surface does not appear to affect the biological activity of the p75 protein.

### Thermal and pH stability of the spore displayed CotG-p75

**Fig. 3:**
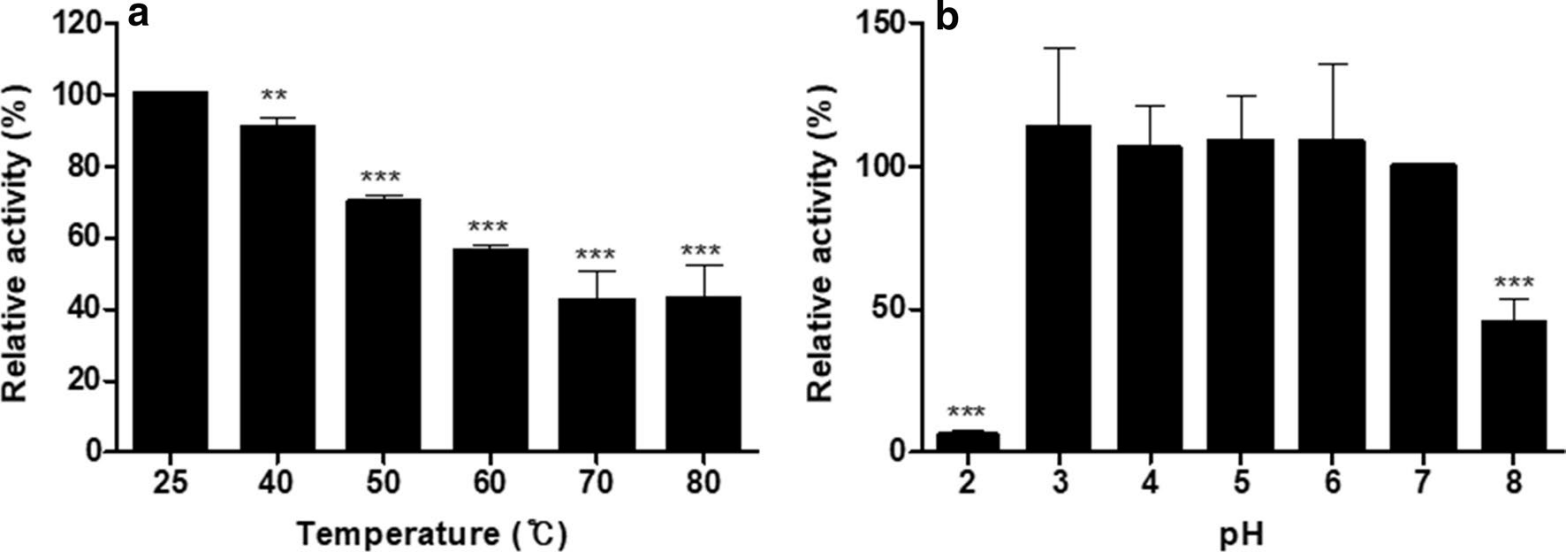
Relative peptidoglycan hydrolase activity of CotG-p75 after heat (**a**) and pH (**b**) treatments. Relative activity was calculated by defining its activity at 25 °C and pH 7 as 100%. All tests were performed in triplicate, and the data are presented as mean ± standard deviation. Statistical analysis was performed by an unpaired two-tailed *t*-test. Asterisks indicate a significance difference from the control (*p < 0.05, **p < 0.01, ***p < 0.001)

To investigate the stability of CotG-p75, its PGH activity was determined under various temperature and pH conditions. The PGH activity of CotG-p75 measured at room temperature (25 °C) and pH 7 was used as a control. As shown in Fig. 3a, the PGH activity of the CotG-p75 decreased gradually as the temperature increased from 40 °C to 80 °C. The activity of CotG-p75 was maintained at more than 90% at 40 °C and 70% and 56% at 50 °C and 60 °C, respectively. The CotG-p75 appears to be relatively thermostable and could maintain activity above 40% even after heat treatment at 80 °C.

The pH stability of CotG-p75 was also tested after treatment at various pHs (2, 3, 4, 5, 6, 7, and 8) for 15 min. As shown in Fig. 3b, the activity of CotG-p75 remained stable in the range of pH 3–7 (*p *> 0.05). However, the activity of CotG-p75 was significantly reduced to 6% at pH 2, a strong acidic condition, and to 46% at pH 8, a relatively weak alkaline condition.

### Antibacterial effect of CotG-p75 against *L. monocytogenes*

**Fig. 4:**
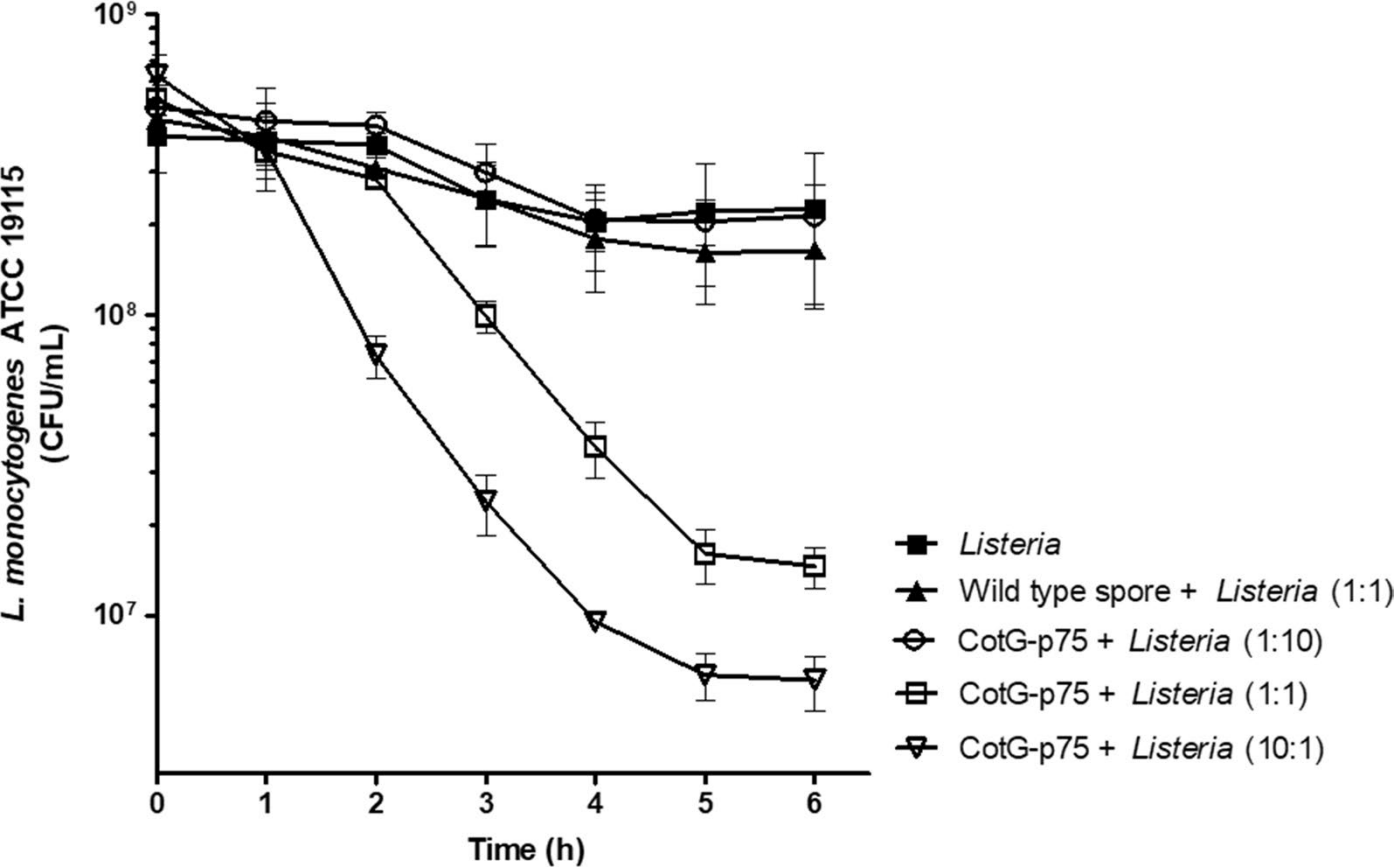
Antibacterial effect of CotG-p75 against *L. monocytogenes*. Cells were treated with different concentrations of CotG-p75 in PBS at 37 °C. The control groups were treated with no spores (closed square) or wild-type spores (closed triangle). The number of viable cells was measured at intervals of 1 h during the 6 h incubation. All tests were performed in triplicate, and the data are presented as mean ± standard deviation. Statistical analysis was performed by an unpaired two-tailed *t*-test

To evaluate the antibacterial effect of CotG-p75, a time-kill assay was conducted against *L. monocytogenes*. The CotG-p75 spores at concentrations of 10^7^, 10^8^, and 10^9^ CFU/mL were mixed with a constant number of *L. monocytogenes* cells (10^8^ CFU/mL) and the viability of the cells was analyzed (Fig. 4). After 6 h in the control group with PBS or wild-type spores, the viable number of *L. monocytogenes* cells decreased by 0.24 log and 0.44 log, respectively, from the initial cell count. When the ratio of CotG-p75 to *L. monocytogenes* was 0.1, the viable cell count of *L. monocytogenes* decreased by 0.36 log, which was not different from the control groups. However, when the CotG-p75/*L. monocytogenes* ratios were 1 and 10, the viable cell counts of *L. monocytogenes* tended to decrease rapidly over time and decreased by 1.55 and 2.02 logs after 6 h, respectively. Although the number of p75 proteins displayed on each of the spore’s surface was not measured in this study, the viable count of *L. monocytogenes* clearly decreased as the amount of CotG-p75 increased. The decrease in viable cell number measured by the time-kill assay was roughly confirmed by light microscopy and is presented in Additional file 1: Fig. S1.

### Scanning electron microscopy image of *L. monocytogenes* treated with CotG-p75

**Fig. 5:**
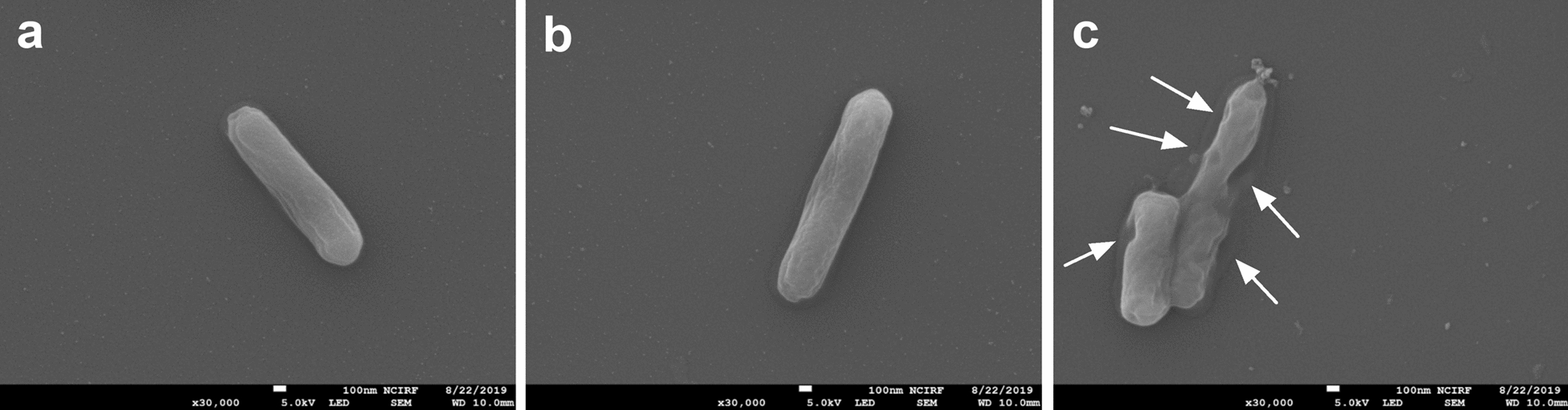
SEM images of *L. monocytogenes* untreated (**a**) and treated with wild type spores (**b**), and CotG-p75 (**c**) in PBS for 6 h at 37 °C. Arrows indicate damaged cell surfaces

In order to confirm whether the antibacterial effect of CotG-p75 was caused by PG degradation, the morphological changes of *L. monocytogenes* treated with CotG-p75 were observed using SEM (Fig. 5). When *L. monocytogenes* cells were exposed to PBS (Fig. 5a) and wild-type spores (Fig. 5b), the surface of *L. monocytogenes* did not show any significant changes. However, as shown in Fig. 5c, *L. monocytogenes* cells treated with CotG-p75 for 6 h exhibited a dented and deformed surface as compared with the controls. In Fig. 5c, it is not clear whether the damaged cells are alive or dead. However, combining this result with the time-kill assay and light microscopy results, the p75 protein displayed on the spore surface appears to have antibacterial activity mediated by its effects on the integrity of the cell wall of *L. monocytogenes*.

## Discussion

Many concerns have been raised about the risks of probiotics such as bacterial infections and sepsis. These risks are not a problem for healthy people but can be fatal for infants, the elderly, and immunocompromised patients (Boyle et al. 2006). To minimize the risks of bacterial probiotics, proteins secreted by probiotic strains have been proposed as safer and more effective alternatives (Sánchez et al. 2010).

Among the many secreted proteins, p75 is one of the well-known soluble proteins produced by LGG and its probiotic actions have been widely studied. In particular, isolated p75 was more effective than whole bacteria (live or heat-killed LGG) in maintaining the homeostasis of intestinal epithelial cells (Seth et al. 2008). However, there are practical limitations to using the purified proteins as a probiotics alternative due to cost and stability issues. In that regard, we chose *B. subtilis* spores as the vehicle for the probiotic protein p75 to develop new probiotics with improved safety and stability.

Recently, spore surface display technology has been extensively used in the food industries due to its robust resistance in a harsh environment (Zhang et al. 2019). Probiotics need to be highly resistant to various stress conditions, such as manufacturing processes, distribution, storage, and the gastrointestinal transit after ingestion. Therefore, the application of spore surface display technology to probiotics development is expected to improve the stability of probiotics even under harsh conditions.

In this study, we successfully displayed p75 on the surface of *B. subtilis* spores and verified its stable expression by measuring PGH activity at a wide range of temperatures and pH. Our results suggested that CotG-p75 is a probiotics candidate with stability under a broad spectrum of thermal and pH conditions. In addition, CotG-p75 showed antibacterial activity against *L. monocytogenes* but had no effect against *Staphylococcus aureus* (Additional file 1: Fig. S3).

p75 is known to have homology with PGH, which catalyzes the cleavage of the sugar or amino acid chain of PG (Bäuerl et al. 2010; Claes et al. 2012). PGHs form a very diverse group of enzymes and are involved in the growth and division of bacteria, such as cell wall expansion, PG turnover, and daughter cell separation (Vollmer et al. 2008). Since PGH has bacteriolytic activity, it has been studied as a potential antimicrobial agent to replace conventional antibiotics for prevention of bacterial infection and the development of multi-drug resistance (Parisien et al. 2008). For example, the lysostaphin of *Staphylococcus simulans* has been extensively studied and is noted for its cell wall lytic effect against *Staphylococcus aureus* (Baba and Schneewind 1996). Zoocin A of *Streptococcus zooepidemicus* 4881 and millericin B of *Streptococcus milleri* NMSCC061 exhibited antibacterial activity against the *S. aureus* group (Simmonds et al. 1996) and a wide range of Gram-positive bacteria, respectively (Beukes et al. 2000). Among the probiotics, the recombinant cell wall hydrolase from *Lactobacillus paracasei* NRL B-50314 produced by *Bacillus megaterium* showed an antibacterial effect against *Lactobacillus lactis* LM 0230 (Liu et al. 2015). The PGH of probiotic *Pediococcus acidilactici* NCDC 252 also showed cell wall lytic activity against *Micrococcus lysodeikticus* and *Bacillus cereus* (Gandhi et al. 2020).

Although many studies have reported the antibacterial effects of PGH on Gram-positive bacteria, not all PGHs have antibacterial activity. The antimicrobial activity of bacterial PGH has a substrate specificity depending on the enzyme source and it exhibits a narrow spectrum that functions against only certain bacterial species or subspecies (Parisien et al. 2008).

The p75 proteins of *L. casei* and *L. rhamnosus* are known to belong to a large family of secreted cell wall proteins with PGH activity (Bäuerl et al. 2019). The purified p75 protein from *L. casei* has no significant lytic effect against *Enterococcus faecalis*, *L. monocytogenes*, or *S. aureus* (Bäuerl et al. 2019). This result is somewhat different from the results of this study, in which CotG-p75 showed antibacterial effects against *L. monocytogenes*. In *L. casei* and *L. rhamnosus*, the C-terminal amino acid sequence of the p75 protein is well conserved between the two species, but the sequences of the N-terminus region are quite different (Bäuerl et al. 2010). In the previous studies, truncation of the cell wall binding domain, which is usually located in the N-terminus of PGH, reduced its autolytic activity (Gargis et al. 2010; Osipovitch and Griswold 2015). Therefore, the observed differences in the antibacterial activity of p75 protein against *L. monocytogenes* may be due to the differences between the PGHs produced by the two species, *L. casei and L. rhamnosus*. In addition, in the case of CotG-p75, the N-terminus of the p75 was directly connected to the C-terminus of the CotG protein, and thus the possibility that the structure of the p75 was affected cannot be excluded.

In conclusion, the p75 protein of *L. rhamnosus* GG was successfully displayed on *B. subtilis* spores with CotG as an anchoring motif. Stable expression of CotG-p75 was demonstrated with PGH activity in a wide range of temperature and pH conditions. The CotG-p75 showed antibacterial activity against *L. monocytogenes*, probably due to the cell wall hydrolytic activity of p75. The results suggest that the spore surface-displayed CotG-p75 may be a potential antimicrobial agent against *L. monocytogenes*. However, developing CotG-p75 as a new probiotics candidate requires additional research and evaluation of its probiotic activity.

## Supplementary information

**Additional file 1.** Additional figures.

## Data Availability

The datasets supporting the conclusions of this article are included within the article and its additional files.

## Abbreviations

ATCC: American type culture collection
CFU: Colony forming units
LGG: *Lactobacillus rhamnosus* GG
PBS: Phosphate-buffered saline
PCR: Polymerase chain reaction
PG: Peptidoglycan
PGH: Peptidoglycan hydrolase
SDS: Sodium dodecyl sulfate
SEM: Scanning electron microscopy
