# Supplementary material for: Surface display of p75, a Lactobacillus rhamnosus GG derived protein, on Bacillus subtilis spores and its antibacterial activity against Listeria monocytogenes
Source: AMB Express. 2020 Aug 8;10:139. doi: 10.1186/s13568-020-01073-9 (PMC7415045; doi:10.1186/s13568-020-01073-9)
Supplement: Supplementary file 1 — Additional file 1. Additional figures. [file 13568_2020_1073_MOESM1_ESM.docx]

**Journal name:**

AMB express

**Manuscript title:**

Surface display of p75, a *Lactobacillus rhamnosus* GG derived protein, on *Bacillus subtilis* spores and its antibacterial activity against *Listeria monocytogenes*

**The names of the authors:**

Soo Ji Kang^1^, Ji Su Jun^1^, Jeong A Moon^1^ and Kwang Won Hong^1,^*

**The affiliation and address of the authors:**

^1^ Department of Food Science and Biotechnology, College of Life Science and Biotechnology, Dongguk University, Goyang-si 10326, Republic of Korea

*** Corresponding author:** Kwang Won Hong ^(^E-mail address: [hkwon@dongguk.edu](mailto:hkwon@dongguk.edu))

**
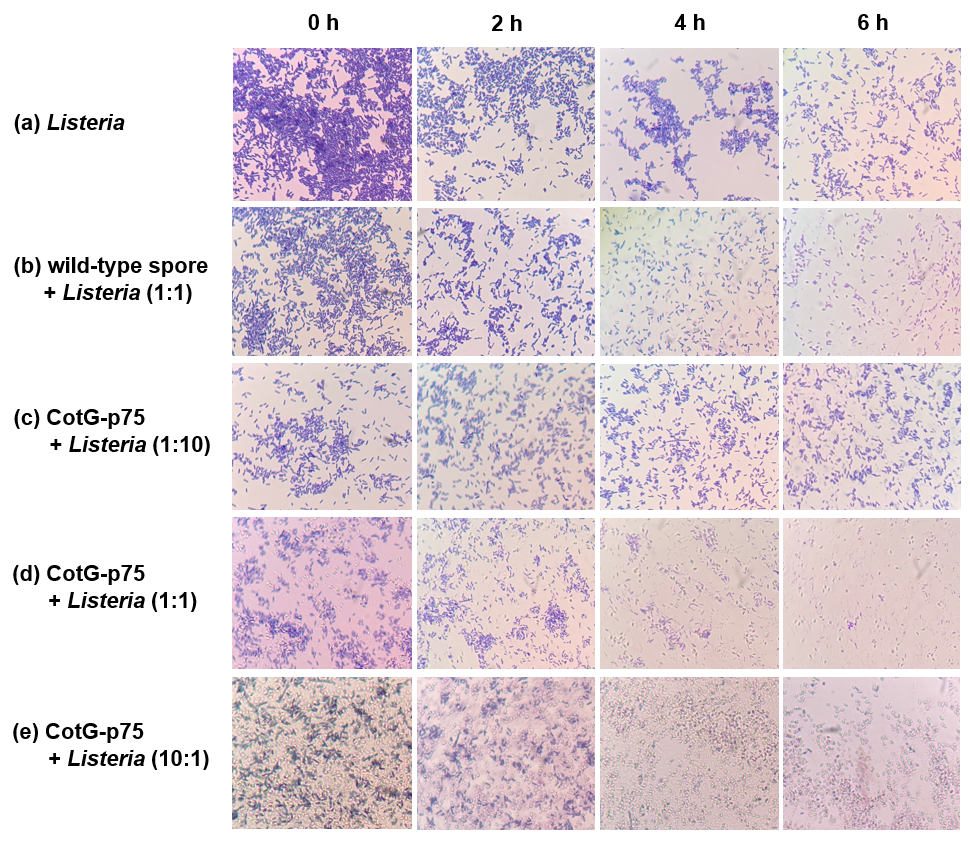
Fig. S1** Light microscope images of *L. monocytogenes* cells treated with no spores (a), wild-type spores (b), and CotG-p75 (c–e) for 0, 2, 4, and 6 h at 37℃ in PBS. In panels (c–e), CotG-p75 and *L. monocytogenes* cells were mixed at a ratio of 1:10 (c), 1:1 (d) and 10:1 (e). Gram-stained bacterial cells were observed under oil immersion (1000×). Purple-stained rod-shaped images are *L. monocytogenes*, while unstained round images are spores (especially in panel e).

**
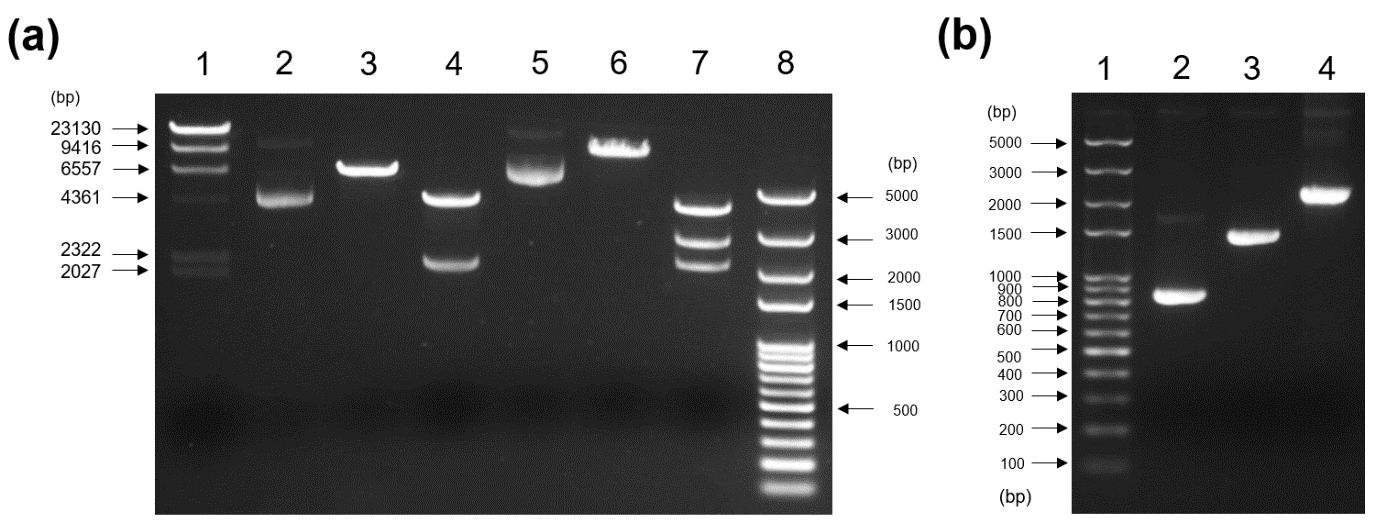
Fig. S2** Identification of the recombinant plasmid pUB19-*cotG*-*p75* by restriction enzyme analysis (a) and PCR amplification (b). In panel (a): Lane 1, λ DNA/*Hin*dⅢ marker; Lane 2, pUB19 plasmid DNA; Lane 3, pUB19 linearized by *Bam*HⅠ (6.4 kb); Lane 4, pUB19 digested by *Eco*O109Ⅰ (4.3 and 2.1 kb); Lane 5, pUB19-*cotG*-*p75* plasmid DNA; Lane 6, pUB19-*cotG*-*p75* linearized by *Mlu*Ⅰ (8.8 kb); Lane 7, pUB19-*cotG*-*p75* digested by *Eco*O109Ⅰ (4.5, 2.2, and 2.1 kb); Lane 8, 100 bp plus Ⅱ DNA ladder (TransGen Biotech, Beijing, China). In panel (b): Lane 1, 100 bp plus Ⅱ DNA ladder; Lane 2, the amplified *cotG* gene with the CotG-F/R primer (0.87 kb); Lane 3, the amplified *p75* gene with the P75-F/R primer (1.44 kb); Lane 4, the amplified *cotG-p75* gene with the CotG-F/P75-R primer (2.26 kb).


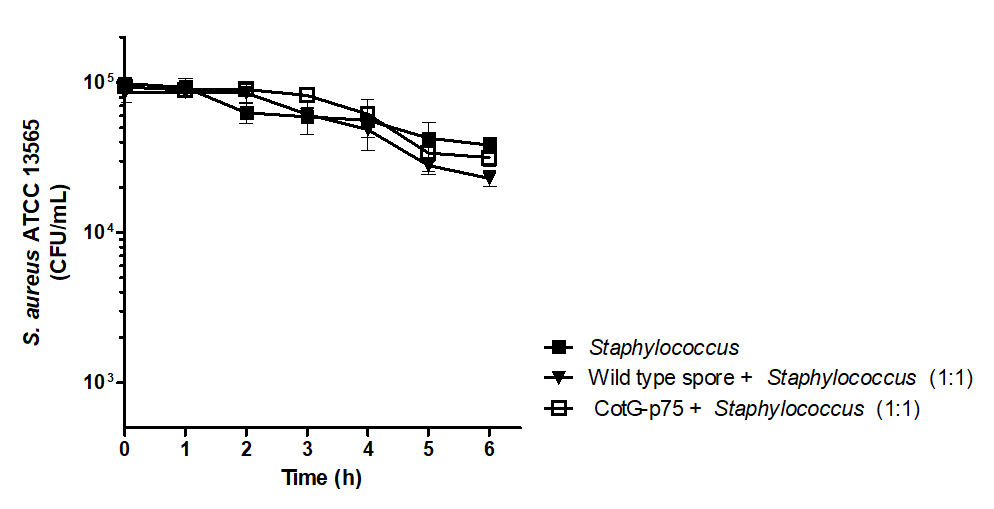


**Fig. S3** Antibacterial test of CotG-p75 against *S. aureus* ATCC 13565. Cells were treated with CotG-p75 in PBS at 37℃. The control groups were treated with no spores (■) or wild-type spores (▼). During the incubation, 0.1 mL of each culture was taken at 1 h intervals, diluted as indicated, and then plated on Baird-Parker medium for *S. aureus*. All tests were performed in triplicate, and the data are presented as mean ± standard deviation. Statistical analysis was performed by an unpaired two-tailed *t*-test.
